# Effect of a novel stretching technique on shoulder range of motion in overhead athletes with glenohumeral internal rotation deficits: a randomized controlled trial

**DOI:** 10.1186/s12891-021-04292-8

**Published:** 2021-04-30

**Authors:** Omar Gharisia, Everett Lohman, Noha Daher, Alan Eldridge, Amjad Shallan, Hatem Jaber

**Affiliations:** 1 Department of Physical Therapy, School of Allied Health Professions, Loma Linda University, Loma Linda, CA USA; 2 Department of Allied Health Studies, School of Allied Health Professions, Loma Linda University, Loma Linda, CA USA; 3 Department of Physical Therapy, Faculty of Applied Medical Sciences, The Hashemite University, Zarqa, Jordan; 4 Department of Physical Therapy, College of Rehabilitative Sciences, University of St. Augustine for Health Sciences, Austin, TX USA; 5 Department of Physical Therapy, College of Health and Human Development, California State University, CA Northridge, USA

**Keywords:** Glenohumeral internal rotation deficits, Novel stretch technique, Overhead athletes

## Abstract

**Background:**

The cross-body and the modified sleeper stretch have been used to improve posterior shoulder soft tissue flexibility and to increase glenohumeral joint internal rotation (GHJ IR) in overhead athletes. However, due to the inability to stabilize patient’s scapula and control GHJ rotation with the cross-body stretch and the potential for subacromial impingement or symptoms’ aggravation with the modified sleeper stretch, a new stretching technique (Passive Glenohumeral Internal Rotation with Clam Shell Bridging) was developed as an alternative to these commonly used stretches that may allow for greater stability of the scapula without reproducing symptoms. Thus, the current study aimed to examine and compare a novel stretching technique to the traditional modified sleeper stretch to determine the effect on glenohumeral IR range of motion (ROM) and self-reported pain in overhead athletes with glenohumeral internal rotation deficits (GIRD).

**Methods:**

Forty-two overhead athletes with GIRD [mean age 25.9 ± 2.6 years, 20 males and 22 females] participated in this study. Participants were randomly assigned into either novel stretching group or modified sleeper stretching group. IR ROM was measured with a digital inclinometer before, immediately, and at week 4 post intervention, while pain was measured with Numeric Pain Rating Scale before and at week 4 post intervention.

**Results:**

There was no significant group by time interaction effect for IR ROM (*p* = 0.27); however, there was a significant change over time (*p* < 0.001, η^2^ = 0.77). Both groups demonstrated a significant increase in IR from baseline to immediate and week 4, and from immediate to week 4 (*p* < 0.001). There was a significant group by time interaction for pain intensity (*p* < 0.001, η^2^ = 0.72). Results showed a significant reduction in pain intensity over time in the novel group (*p* = 0.001, d = 2.18), but not in the traditional group (*p* = 0.231, d = 0.46).

**Conclusion:**

Both stretches appear to be effective at improving IR ROM in overhead athletes with GIRD. However, the novel stretching might be more effective at reducing shoulder pain and thus may be more appropriate for symptomatic patients.

**Trial registration:**

Prospectively registered in February 6, 2017 under Clinical Trial Registry # NCT03044236.

## Background

The shoulder complex is the most commonly injured body part in overhead and throwing athletes, accounting for 30% of all injuries [1, 2]. With the growing interest and participation in overhead sports such as baseball, tennis, volleyball, squash, swimming or water polo, the incidence of shoulder pain and/ or injuries continues to increase [3–5]. In fact, more than half of the general population will experience shoulder pain at one time or another in their lives [6]. Moreover, about 20% of all disability costs from musculoskeletal abnormalities are related to shoulder problems [7]. Compromised shoulder movement due to pain or injury can cause substantial disability and affect a person’s ability to carry out daily activities and restricts sport participation.

It has been reported that individuals who frequently participate in overhead throwing sports demonstrate an altered mobility patterns in the throwing shoulder as compared to the non-throwing shoulder [8]. These adaptive mobility patterns usually present as excessive external rotation (ER) and decreased internal rotation (IR) of the throwing shoulder [9]. This loss of IR range (15°-25°) has been defined as Glenohumeral Internal Rotation Deficit (GIRD) [9–11] and has been linked to adaptive structural changes in the soft tissue (i.e. capsuloligamentous or muscular tightness) and/or osseous tissue (i.e. humeral retroversion) of the glenohumeral (GH) joint as result of the extreme demands and the excessive overload of repetitive overhead activities [8].

Posterior inferior capsular and rotator-cuff tightness have been suggested as the main contributing factors to the loss of GH IR range of motion (ROM) for most athletes [8, 9]. The repetitive overhead motions which usually involve excessive ER with a maximally abducted position (cocking phase) and the subsequent phases of acceleration/deceleration and follow-through place a high amount of stress on the static and dynamic stabilizers of the shoulder, including the rotator cuff, joint capsule, and labrum. This repetitive overuse load will ultimately lead to a failure in these structures to absorb arm energy, predisposing them to injury, which generates pain and posterior shoulder tightness [8, 9]. If left uncorrected, this increased tightness in the posterior structures may cause the humeral head to translate anterosuperiorly during elevation and IR or posterosuperiorly during maximal ER leading to subacromial impingement and/or Type II Superior Labral Anterior to Posterior (SLAP) lesions [12–15]. In a study by Burkhart et al. [16], pitchers were shown to have a loss of IR in the shoulder with the SLAP lesion compared to the non-injured shoulder. Overhead athletes with shoulder internal impingement were also found to have a reduction in GH IR as a result of posterior shoulder tightness [10]. Evidence has suggested that overhead athletes who demonstrate a limitation of ≥25° in shoulder IR are at 4 times higher risk of shoulder or elbow pain and injury while those with a limitation of ≥10° are at approximately 2 times higher risk [12].

Stretching has been proposed as an effective approach for the management of GIRD, restoring shoulder ROM, and reducing the incidence of shoulder injury and muscle soreness [15, 17]. There are a number of stretching techniques, such as cross-body or horizontal adduction stretches that have been used to improve GHJ ROM [18]. Manske et al. [19] examined the effect of 4 weeks of cross-body stretching alone versus cross-body stretching and posterior GHJ mobilizations on GHJ IR ROM, and reported similar improvement in GHJ IR for both interventions. However, the inability to stabilize the patient’s scapula and control glenohumeral joint rotation while performing the cross-body stretch has made this technique less favorable [20, 21]. Nonetheless, a newer stretching technique, the Sleeper Stretch, has been adapted and commonly recommended by clinicians to restore the Glenohumeral Joint (GHJ) IR ROM. Burkhart et al. [15] and Launder at al [20] examined the effect of the Sleeper Stretch on shoulder ROM and found that the sleeper stretch group improved significantly in IR ROM compared to the control stretching group. However, a potential for subacromial impingement or aggravation of symptoms with the traditional and the modified sleeper stretch has been documented [21, 22].

**Fig. 1:**
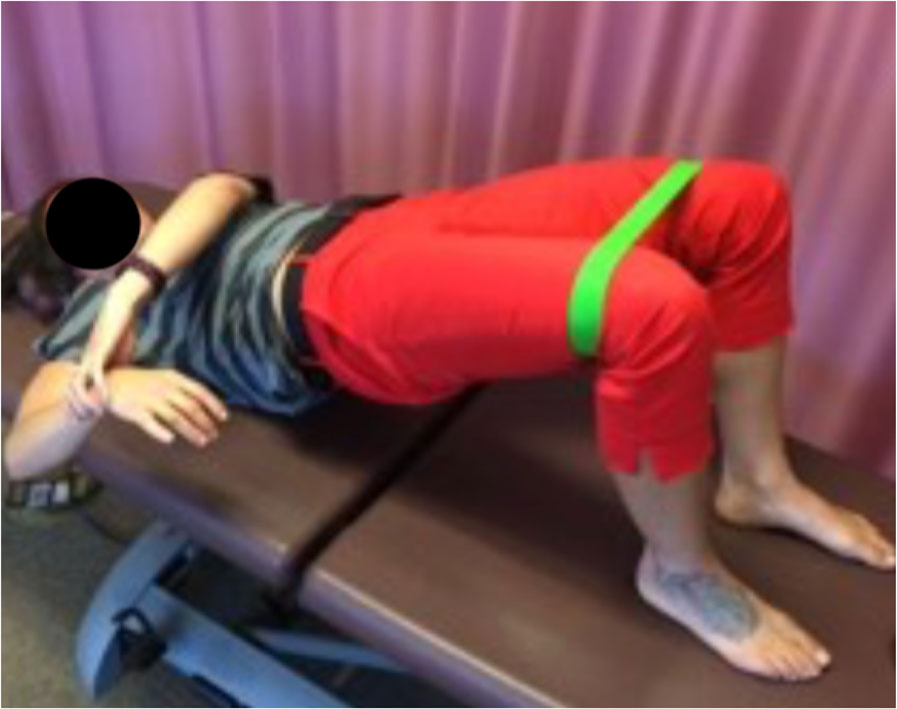
Passive Glenohumeral Internal Rotation with Clam Shell Bridging

Although both static stretching techniques (sleeper and cross-body stretches) have been the primary stretches for restoring GHJ IR, the lack of scapular stabilization or the aggravation of symptoms remain a concern for clinicians that prescribe these stretching techniques to patients and clients with GIRD. Given this, we sought to justify a new stretching technique as an alternative to the commonly used stretching techniques that may allow for greater stability of the scapula without aggravating or reproducing symptoms. Thus, the novel technique could provide favorable outcomes. The novel technique is performed while the subject is in a resisted bridging position (Fig. 1). Bridging was incorporated into the new technique as an approach to minimize the contact area to mainly the upper back and scapula, thus more pressure is placed on the scapula. That way, scapular movement might be very limited allowing for a proper stretching of the external rotators.

With the growing evidence regarding the effect of stretching on GIRD, it is important that this novel technique be empirically evaluated, accurately described, and reported to the clinical and scientific communities. Therefore, the current study aimed to examine and compare a novel stretching technique to the traditional modified sleeper stretch to determine the effect on GHJ IR ROM and self-reported pain in overhead athletes with GIRD.

## Methods

### Trial design

This was a parallel-design 2-arm, assessor-blinded randomized controlled clinical trial. IR ROM was assessed at baseline, immediately post intervention, and at week 4 post intervention. Pain intensity was assessed at baseline and at week 4 post intervention. The study’s protocol was in compliance with the CONSORT guidelines.

### Participants

A sample of 42 physically active adult athletes (20 males, 22 females) voluntarily participated in this study. Participants were recruited by flyers, emails, referrals, and word of mouth from a college campus and a community in Sothern California. All participants read and signed a written informed consent approved by the Institutional Review Board of Loma Linda University prior to participation. Eligible participants met the following inclusion criteria: 1) between 18 and 45 years of age; 2) performed overhead sports activities, such as volleyball, tennis, water polo, squash, baseball, swimming, or lifting as a major activity of interest; 3) participated in local and regional sport competitions 4) displayed a 10° or greater of between-shoulder difference in IR ROM (dominant versus non-dominant); and 5) with or without pain during shoulder activities. These criteria were selected based on a recent systematic review, which suggested that a lower threshold ROM deficit, based on a weighted mean GIRD of 13.8° among all injured athletes, should be utilized if the goal is to improve ROM and prevent injuries in these athletes [23]. Subjects were excluded if they were: 1) still recovering from previous surgery of the shoulder and elbow complex in the past 3 months; 2) currently receiving medical intervention for the shoulder; or 3) have any critical medical condition. All experimental procedures were conducted in the orthopedic laboratory at Loma Linda University, Department of Physical Therapy from April 2017 to November 2018.

### Instrumentation

A digital inclinometer (MicroFET3, Draper, UT) was used to measure GH IR and ER ROMs. This instrument has been shown to have a high intra-rater and inter-rater reliability [Intraclass correlation coefficient (ICC) = 0.87 and ICC = 0.93, respectively] [24]. Subsequently, a two-pound pressure, via an electronic push/pull dynamometer (Baseline Electronic Push/Pull Dynamometer - MicroFET3, Draper, UT), was applied by the same examiner at the end of available passive range of motion prior to taking a ROM measurement in order to standardize the shoulder internal and external rotation values. Pain was measured using the Numeric Pain Rating Scale (NPRS). NPRS has been shown to be valid, reliable, and appropriate for use in clinical practice [25].

### Procedures and intervention

**Fig. 2:**
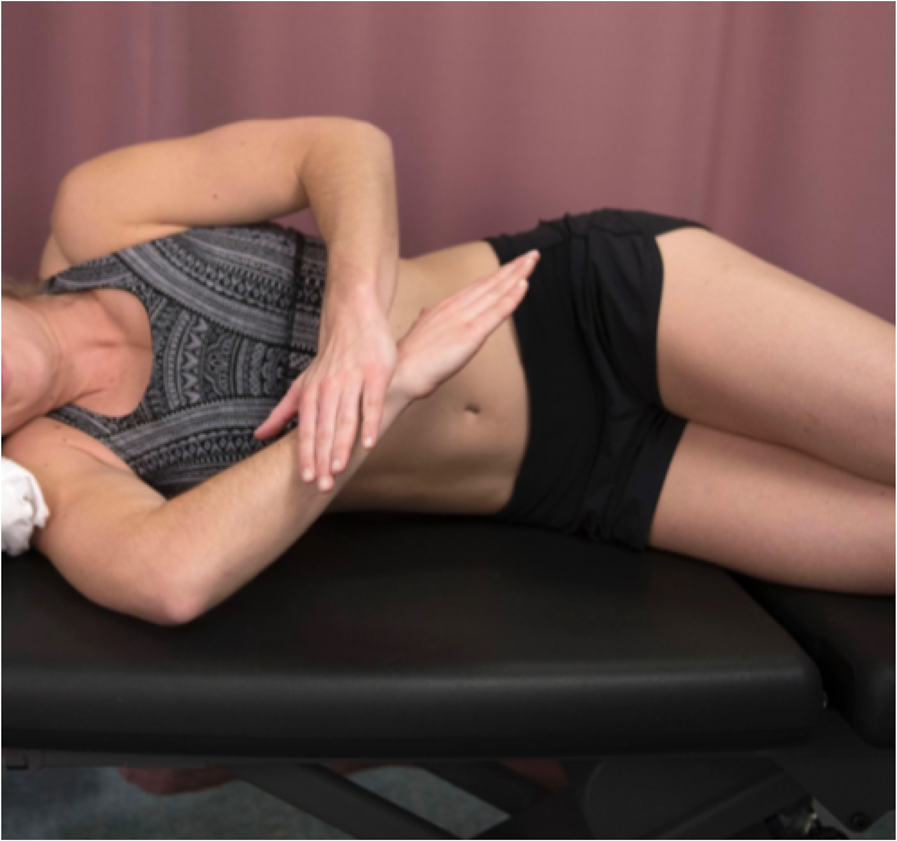
Modified Sleeper Stretch: Passive Glenohumeral Joint Internal Rotation

Forty subjects who fulfilled the inclusion criteria were recruited. After stratification, participants were randomly assigned to one of the 2 intervention groups. Group A comprised the modified sleeper stretch (MSS) group (*n* = 22) (Fig. 2) while Group B comprised the novel stretch (NS) group (*n* = 20) (Fig. 1). Group assignment was conducted by an independent person using a random number generator and concealed in sealed envelopes from all personnel involved in screening before randomization. Post randomization, objective measures’ assessors were blinded to group assignment. Participants were taught their assigned home-based stretching program by the investigator who explained the stretching technique to each participant. All participants were asked to demonstrate the stretch to ensure mastery. Additionally, to ensure and facilitate compliance, participants were provided a written sheet with detailed instructions and demonstrative pictures of the stretch. A log sheet was provided to monitor the number of stretches per week. Participants were also contacted weekly through phone calls and text messages as a reminder to minimize lack of compliance. All participants performed 4-weeks of stretching protocol, three times a week, with three repetitions, holding each stretch for 30 s, with 30 s rest between repetitions [26].

Participants in Group A were instructed to perform the MSS in a side-lying position, on the side to be stretched, with the elbow flexed to 90°, shoulder elevated to 90°, and the trunk slightly rolled posteriorly 20^0^ to 30^0^ to place the shoulder in the scapular plane as passive IR is performed (Fig. 2). Participants were instructed to allow the stretched shoulder to naturally fall into IR to the end ROM where resistance could be felt. Participants were then instructed to use the other hand to push the stretched shoulder into further IR to the point of mild stretch discomfort by applying pressure at the area of the wrist joint.

Participants in Group B were instructed to perform the NS in a supine position. Participants placed a resistance band around their knees and were instructed to open their knees. Participants were asked to bridge as high as possible, with the elbow flexed to 90° and shoulder abducted to 90° (Fig. 1). The bridging maneuver shifts the body weight superiorly pinning the medial border of scapula against the thorax without directly compressing or restricting posterior shoulder structures. It is proposed that this position will allow greater freedom of motion with less discomfort. While maintaining this position, participants were asked to contract or “squeeze” their gluteal muscles and perform the stretching by actively internally rotating their shoulder to the end of ROM. Participants used the other hand to push to the point of mild stretch discomfort and simultaneously maintain contraction while progressing the stretch.

Shoulder IR was measured by positioning the participant in a side lying position, with the elbow flexed to 90° and shoulder abducted to 90°. Participants were instructed to allow the shoulder to naturally fall into internal rotation to the end ROM where resistance was felt. At this point, an electronic push/pull dynamometer was applied on the same location to the distal forearm at the end range of motion. Before recording the ROM measurement, the physical therapist applied consistent pressure (2 pounds) in an attempt to standardize and quantify the passive force to ensure reliable and valid ROM measurements. The digital inclinometer was then aligned along the ulna of the stretched arm and three trials of the internal rotation ROM measurements were taken, averaged and used for analysis.

For subjects experiencing shoulder pain, the intensity of pain was measured using the NPRS. An experienced physical therapist provided a detailed explanation regarding the NPRS prior to the administration of the NPRS questionnaire to ensure better understanding. All participants had to complete the NPRS questionnaire at baseline and post 4-weeks.

### Statistical analyses

A sample size of 42 participants was estimated using a moderate effect size for the group x time interaction (partial η^2^ = 0.06) [27], level of significance (α = 0.05), power of 0.80, and a 35% dropout.

Data was summarized using mean and standard deviation for quantitative variables and counts (%) for qualitative variables. The normality of continuous variables was examined using Shapiro Wilk’s test and Q-Q normality plots. The distribution of the participants’ characteristics by study group was evaluated using chi-square for qualitative variables, independent t-test for quantitative variables and Mann-Whitney test for ordinal variables. A 2-group _X_ 3-time points (baseline, week 1, and week 4) mixed factorial analysis of variance (ANOVA) was used to examine changes in mean GH IR ROM by study group over time. For pain intensity, 2-group × 2 time points (baseline vs. week 4) mixed factorial ANOVA was used.

The primary analysis included a comparison between groups using the group x time interaction effect. If the interaction was statistically significant, change from baseline was compared between groups at each follow-up time point using an independent t-test. If the results were not statistically significant, the between-groups comparison was considered not statistically significant at any time point. However, Bonferroni post hoc test was conducted on the combined groups only if the main effect of time was significant in the mixed factorial ANOVA.

The secondary analysis included testing of change from baseline at each time point within-groups using one-way repeated measures ANOVA for ROM or paired t-test for pain intensity. If the results of the test were statistically significant, post hoc comparisons using Bonferroni test was conducted on each study group separately. Partial Eta Squared (η^2^) was reported for significant differences in ANOVA and categorized as [0.01 small, 0.06 medium, and ≥ 0.13 large], while Cohen’s d effect size was used for independent t-test and categorized as [0.2 small, 0.5 medium, and ≥ 0.8 large] [28]. The level of significance was set at *p* ≤ 0.05. Statistical analysis was performed using IBM SPSS Software version 25 for Windows (Chicago, IL, USA).

## Results

**Fig. 3:**
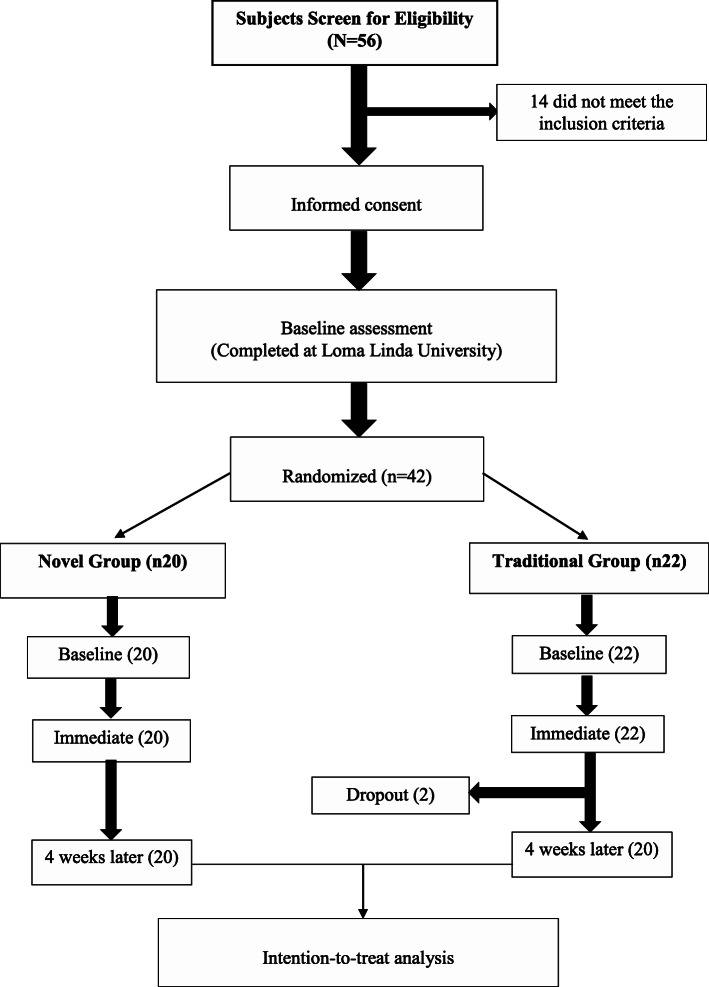
Flow diagram of participants’ recruitment and retention

Forty-two participants with mean age 25.9 ± 2.6 years old and body mass index (BMI) of 19.0 ± 4.2 kg/m^2^ were recruited. Two participants dropped out from the MSS group following week 1 of intervention with unknown reasons, and 40 completed the study (Fig. 3). Fifty-five percent of the participants were females (*n* = 22). The mean IR deficit was 15.6 ± 5.5. Nonetheless, the analysis was performed on all available data from all randomized participants according to the intention-to-treat principle (20 NS and 22 MSS). The compliance rate of home-stretching program was 100% for both groups. In addition, none of the participants reported receiving any outside care during the study period. The distribution of the continuous variables was approximately normal. There were no significant differences in demographic and baseline outcome measures between the two study groups (*p* > 0.05), Table 1. None of the participants reported any harm or adverse effects following interventions.

**Table 1:** Mean (SD) of General Characteristics and Baseline Outcomes (*N* = 42)

	Novel Stretching (n_**1**_ = 20)	Traditional (n_**2**_ = 22)
**Female; n (%)**	11 (55)	11 (50)
**Age (years)**	26.0 (2.5)	25.9 (2.6)
**BMI (kg/m**^**2**^**)**	27.3 (4.5)	25.1 (3.5)
**GIRD (degree)**	15.8 (5.1)	15.4 (5.3)
**Compliance Rate (%)**	100 (0)	100 (0)
**Baseline Pain**	4.43 (1.4)	2.57 (0.54)
**GHIR_ROM (degree)**	49.2 (12.7)	49.6 (15.7)

*Abbreviations*: *SD* Standard Deviation, *GHIR_ROM* Glenohumeral Internal Rotation Range of Motion

Results of the mixed factorial ANOVA are displayed in Table 2. There was no significant group by time interaction effect for GH IR ROM (*p* = 0.27); however, there was a significant change over time (*p* < 0.001, η^2^ = 0.77) for the combined groups. Bonferroni post hoc comparison revealed that IR ROM significantly increased from baseline to immediate (49.4 ± 14.1 vs. 63.1 ± 13.2, *p* < 0.001) and week 4 (49.4 ± 14.1 vs. 70.0 ± 12.7, *p* < 0.001), and from immediate to week 4 (63.1 ± 13.2 vs. 70 ± 12.7, *p* < 0.001). Similar results were obtained after controlling for gender [there was no significant interaction (*p* = 0.25), but there was a significant change over time (*p* < 0.001)].

**Table 2:** Mean (SD) of Glenohumeral Internal ROM (N) by Study Group over Time (*N* = 42)

	Novel Stretching (n_1_ = 20)			Traditional Stretching (n_2_ = 22)			*p*-value over time^*^ (η^2^)	*p*-value (group x time)^*^ (η^2^)
	Baseline	Immediate	4 weeks later	Baseline	Immediate	4 weeks later		
GHIR_ROM^a^	49.2 (12.7)	63.7 (10.3)	72.0 (10.6)	49.6 (15.7)	62.6 (15.8)	68.1 (14.5)	< 0.001 (0.77)	0.27 (0.03)

*Abbreviations*: *SD* Standard Deviation, *GHIR_ROM* Glenohumeral Internal Rotation Range of Motion; *η*^*2*^ Partial Eta Squared

*Mixed Factorial ANOVA, level of significance was set at p ≤ 0.05

^a^Significant difference between baseline & immediate, baseline & week 4, and immediate & week 4 (*p* < 0.001)

For pain intensity, none of those who were asymptomatic reported any pain 4 weeks post-intervention. However, for those who were symptomatic at baseline (7 novel, 7 traditional), there was a significant group by time interaction after controlling for baseline pain (*p* < 0.001, η^2^ = 0.72), Table 3. Results of the independent t-test showed a significant difference between the two groups at week 4 post intervention (*p* = 0.002, d = 2.17), Table 4. Results of the paired t-test showed a significant reduction in pain intensity over time in the novel group (*p* = 0.001, d = 2.18). However, there was no significant change in pain intensity over time for the traditional group (*p* = 0.231, d = 0.46), Table 3. It is note mentioning that though pain analysis was based on a smaller sample size (*N* = 14), post hoc power analysis using the effect size of 0.58 that was found in pain intensity interaction effect, revealed that the observed power was 0.99.

**Table 3:** Mean (SD) of Pain Intensity by Study Group over Time (*N* = 14)

	Novel Stretching (n_1_ = 7)^a^		Traditional Stretching (n_2_ = 7)		*p*-value over time^*^ (η^2^)	*p*-value (group x time)^*^ (η^2^)
	Baseline	4 weeks later	Baseline	4 weeks later		
Pain Intensity	4.43 (1.39)	0.71 (0.49)	2.57 (0.54)	2.00 (1.40)	0.019 (0.38)	< 0.001 (0.72)

*Abbreviations*: *SD* Standard Deviation, *η*^*2*^ Partial Eta Squared

*Analysis of Covariance, level of significance was set at *p* ≤ 0.05

^a^Significant difference between baseline & week 4 (*p* = 0.001)

**Table 4:** Mean (SD) Change of Pain Intensity from Baseline by Study Group (*N* = 14)

	Time Change from Baseline	Novel Stretching (n_1_ = 7)	Traditional Stretching (n_2_ = 7)	Mean Difference (95% CI)	*p*-value*	Effect Size (Cohen’s d)
Pain Intensity	**Week 4**	3.72 (1.70)	0.57 (1.13)	3.14 (1.46, 4.83)	0.002	**2.17**

*Abbreviation*: *SD* Standard Deviation, *CI* Confidence Interval

*Independent t-Test, level of significance was set at *p* ≤ 0.05

## Discussion

### Glenohumeral internal rotation range of motion

The results of the current study suggest that in overhead athletes with GIRD, the NS technique (Passive Glenohumeral Internal Rotation with Clam Shell Bridging) is no more effective than the traditional MSS at improving GH IR ROM. Both groups demonstrated significant increases in IR from baseline to immediate and week 4, and from immediate to week 4. It can be inferred that the increases noted in both groups reflect a real increase in IR taking into consideration the minimal detectable change (MDC_95_) values we obtained. The MDC represents the smallest change in measurement over time that reflects a true threshold change rather than simple measurement error [29]. The MDC_95_ values reported in this study indicated that a change greater than or equal to 9.3° was required to be 95% certain that the change was not due to subject/intertrial variability or measurement error. The changes from baseline to immediate were NS 14.5°, MSS 13° and from baseline to week 4 were NS 22.8°, MSS 18.5°, which exceeded the obtained MDC value. Similarly, Kolber et al. [30, 31] have suggested that a MDC_90_ value of 8°-9° should be considered when interpreting change values over subsequent treatment sessions. However, given that we did not calculate the minimal clinically important difference (MCID), which represents the smallest amount of change that is clinically meaningful as perceived by the patient [32], and that there is no reported reference (threshold) value for MCID in literature, we are uncertain as to what extent the obtained change would be considered clinically meaningful.

Though we did not re-examine whether or not the side-to-side differences in IR still existed following the stretching techniques as we only measured the improvement in the treated dominant side over time, the proportion of those who showed an improvement in IR that reached or exceeded the mean IR deficit of 15.6° was high, 80% (90% in novel vs. 75% in traditional). This indicates that such differences would have been diminished/disappeared post intervention.

Comparing these results with other research findings, however, is difficult because of the identical lack of studies that used/compared the same stretching procedures. However, the clinical merits of these stretching techniques could be established by comparing the outcomes of this study with previous relevant studies. Comparative studies have been carried out to determine effects of various stretching techniques on shoulder ROM deficit. Laudner et al. [20] reported an immediate increase in IR ROM following passive sleeper stretches performed 3 times for 30 s. Bailey et al. [22] and Mine K [33] reported a significantly immediate increase in IR using the cross-body and the sleeper stretches, performed 2 to 5 times for 20s to 1 min. In addition, McClure et al. [34] reported significant improvement in IR following 4 weeks of cross-body stretch and sleeper stretch, with greater increase noted for the cross-body stretch. The findings of the present study are in agreement with the above research studies that showed stretching is an effective method to improve ROM deficits [20, 26, 34]. The observed gains in IR ROM between baseline and immediate (NS 14.5°, MSS 13°) and baseline and week 4 (NS 22.8°, MSS 18.5°) in our study are comparable to those observed in these studies (range 7° to 15°) [33].

### Pain intensity

Only 14 participants (7 novel, 7 traditional) had pain symptoms at baseline. However, a significant reduction (4.43 vs. 0.71, 37% drop) in pain intensity was observed only in the NS group, while the traditional MSS group remained essentially stable. A 30–35% reduction in pain from baseline or a decrease of 1.5 to 1.6 on a 10-point NPRS can be considered significant and was rated as MCID (believed to be meaningful by the patients) [35–37]. Previous research showed symptoms (pain) relief after stretching program in athletes with GIRD and impingement-related shoulder pain [38]. In contrast, McClure et al. [34] reported frequent pain with the sleeper stretch as opposed to the cross-body stretch. However, in order to minimize symptoms of pain aggravation and discomfort associated with the sleeper stretch, we sought to use the MSS [39]. This technique was shown to be as effective as the cross-body stretch at improving IR in persons with GIRD [22]. Our symptomatic participants in the MSS group did not complain or report any aggravation of symptoms during stretching but pain levels remained essentially stable at week 4, which might further support using it over the traditional sleeper stretch. Nonetheless, it is worth mentioning that in Mine K study [22], 7 participants experienced pain during the MSS and therefore were excluded from the study despite the fact that they were asymptomatic before stretching. In contrast, none of the asymptomatic participants in our study reported any pain during or at the end of the protocol. This discrepancy leaves a gap for future research to further investigate and reach a clear conclusion that could help clinicians to make wise decisions that benefit their patients with less adverse effects.

However, given that there was no significant difference between the effects of the MSS and the NS for IR ROM and that the latter was more effective than the MSS at reducing pain, the NS might have better clinical utility, particularly for patients with shoulder pain. In addition, despite previous research studies having shown that the cross-body stretch is more effective than the traditional sleeper stretch [34] and less provocative than both traditional or MSS [22, 34], we chose not to compare the novel technique with the cross-body stretching due to the reported difficulty or inability to stabilize the patients’ scapula [20], which may, at some point, bias the results, especially since our protocol was home-based. However, we still recommend that future studies consider comparing both techniques.

Bridging was incorporated into the new technique as an approach to minimize the contact area, thus more pressure is placed on the scapula without posterior shoulder contact, which might limit scapular movement allowing for a proper stretching of the external rotators. Future studies using pressure sensor analysis system, however, are recommended to validate this hypothetical assumption. Furthermore, the novel stretch is performed in a supine (bridging) position and thus mechanical stress or discomfort to the subacromial tissues may not likely occur as opposed to that with the traditional or modified sleeper stretch [21, 22].

### Limitations

A limitation of this study was the selection of lower threshold GIRD values. However, there is no consensus in literature with respect to the current cut-off values (range, 18°-25°) for GIRD [11, 15, 39] depending on the study design and population. Therefore, in view of maximal protection of the athlete, it is advised that a lower threshold of less than 18° be used [23, 39]. In fact, lower threshold GIRD of > 10° was used by other studies in an attempt to include a wide range of athletes who could potentially benefit from the stretching program [22, 40]. In addition to the above reasons, we selected a lower value because we were interested in demonstrating the possible effectiveness of the novel stretching in general before its application to a more vulnerable population who might be at higher risk of injury. Another limitation is that the inclusion of heterogeneous sample of athletes with different sport activities might have potentially biased our findings. Furthermore, the lack of control group leaves a room for speculation that the prestretching ROM assessment, performed by passively moving the athlete’s shoulder to an end-range, may have resulted in tissue stretching and thereby affected the poststretching (immediate) ROM measurement. However, in a similar study by Laudner et al. [20], the ROM in the true control group (received no intervention) did not differ between the prestretching and poststretching measurements which might indicate that the pre-assessment has no evident or measurable effect on ROM. Additionally, the duration of shoulder pain, for subjects reporting pain at baseline, was not collected at the initial examination to determine the chronicity of pain, and thus the study might have included a mix of subacute and chronic population, and the outcome could be different for these populations in the first place. However, all participants had a duration of more than 2 weeks of shoulder pain. Finally, the lack of follow up to determine which technique would have a lasting effect on the outcomes, and the smaller sample size for the pain variable, which may limit the statistical power. Though the observed post hoc power for pain intensity was shown to be high, we still recommend future studies with a larger sample size and a follow-up to further enhance the generalizability of the study’s findings. Future research should also investigate if adding this active stretch to an overhead athlete’s stretching regimen would have any negative impact on the athlete’s upper extremity strength.

## Conclusion

Both the NS and the MSS appeared to be similarity effective at improving IR ROM in overhead athletes with GIRD. However, the NS may be more effective than the MSS at reducing shoulder pain and thus might be more appropriate for symptomatic patients. Our findings indicate that 3 sets of 30 s stretching bouts might be sufficient in improving IR ROM. Future research should include prospective studies that further assess and validate the effectiveness of the novel stretching as a rehabilitative intervention in a variety of patient population and as a preventive tool for asymptomatic overhead athletes.

### Clinical implications

The findings of the present study help to guide clinicians in the selection of the best available stretching option(s) for improving shoulder IR deficit. The NS and the MSS techniques are effective at improving IR ROM in overhead athletes with GIRD. However, given that the NS is more effective at reducing shoulder pain, the NS might have better clinical utility, particularly for patients with shoulder pain. When shoulder motion is impaired, its restoration should be a key component of rehabilitation to prevent any potential future injuries. The validation of this novel self-stretching technique gives the clinician the option of now giving their clients a self-assisted stretching technique as a part of their home or gym stretching program. This also gives trainers the option of adding this technique to their overhead athletes’ pre-season or mid-season stretching regimen with greater confidence that stretching will not cause or exacerbate symptoms.

## Data Availability

The data that support the findings of this study are presented within the manuscript. However, all relevant raw data are available from LLUH but restrictions apply to the availability of these data, which were used under license for the current study, and so are not publically available. Data are however available from the authors upon reasonable request and with permission of LLUH.

## Abbreviations

ER: External Rotation
IR: Internal Rotation
GIRD: Glenohumeral Internal Rotation Deficit
GH: Glenohumeral
ROM: Range of Motion
GHJ: Glenohumeral Joint
NPRS: Numeric Pain Rating Scale
NS: Novel Stretch
MSS: Modified Sleeper Stretch
BMI: Body Mass Index
MDC_95_: Minimal Detectable Change
MCID: Minimal Clinically Important Difference
SLAP: Superior Labral Anterior Posterior
